# Follow-up of biological reconstruction of epiphysis preserving osteosarcoma around the knee in children: A retrospective cohort study

**DOI:** 10.1097/MD.0000000000033237

**Published:** 2023-03-10

**Authors:** Zhibing Dai, Yachao Sun, Maierdanjiang Maihemuti, Renbing Jiang

**Affiliations:** a Department of Bone and Soft Tissue, Affiliated Tumor Hospital of Xinjiang Medical University, Urumqi, Xinjiang, China.

**Keywords:** biological reconstruction, epiphysis, inactivation bone, liquid nitrogen, osteosarcoma, vascularized fibula

## Abstract

Limb salvage treatment for malignant bone tumors in children includes prosthetic and biological reconstruction. Early function following prosthesis reconstruction is satisfactory; however, there are several complications. Biological reconstruction is another way to treat bone defects. We evaluated the effectiveness of reconstruction of bone defects by liquid nitrogen inactivation of autologous bone with preserving epiphysis in 5 cases of periarticular osteosarcoma of the knee. We retrospectively selected 5 patients with articular osteosarcoma of the knee who underwent epiphyseal-preserving biological reconstruction in our department between January 2019 and January 2020. Femur involvement occurred in 2 cases and tibia involvement occurred in 3 cases, with an average defect of 18 cm (12–30 cm). The 2 patients with femur involvement were treated with inactivated autologous bone by liquid nitrogen with vascularized fibula transplantation. Among the patients with tibia involvement, 2 were treated with inactivated autologous bone with ipsilateral vascularized fibula transplantation and 1 was treated with autologous inactivated bone with contralateral vascularized fibula transplantation. Bone healing was evaluated by regular X-ray examination. At the end of the follow-up, lower limb length, knee flexion, and extension function were evaluated. Patients were followed up for 24 to 36 months. Average bone-healing time was 5.2 months (3–8 months). All patients achieved bone healing with no tumor recurrence and no distant metastasis and all patients survived. The lengths of both lower limbs were equal in 2 cases, with shortening by ≤1 cm in 1 case and shortening by 2 cm in 1 case. Knee flexion was >90° in 4 cases and between 50 and 60° in 1 case. The Muscle and Skeletal Tumor Society score was 24.2 (range 20–26). Inactivation of autogenous bone with the epiphysis preserved by liquid nitrogen combined with vascularized fibula reconstruction for periarticular osteosarcoma of the knee in children is safe and effective. This technique supports bone healing. Postoperative limb length and function, and short-term effects were satisfactory.

## 1. Introduction

Osteosarcoma is the most common primary malignant bone tumor in children and adolescents.^[1]^ Traditionally, the distal femur and proximal tibia were the most common sites for osteosarcomas. In the last century, patients with osteosarcoma had a low survival rate and often underwent amputation. However, with the development of chemotherapy, imaging, and surgical techniques, the 5-year survival rate of osteosarcoma has reached 60 to 70%.^[2]^ Most children with osteosarcoma undergo limb salvage treatment, and there are several methods for reconstruction after tumor resection, including prostheses, large allogeneic bone transplantation, membrane induction technology, bone transfer, and inactivated bone replantation. Prostheses are commonly used to reconstruct bone defects after the resection of malignant bone tumors in children, but there are several long-term complications of prosthesis reconstruction. Epiphyseal destruction in children leads to limb shortening, joint deformity, severe claudication, and compensatory scoliosis.^[3,4]^ Preserving the epiphysis means preserving the growth potential of the patient and preserving the motion function of the knee joint. Autologous bone inactivation and replantation can save costs and match bone defects. This study reviewed 5 patients with osteosarcoma who underwent biological reconstruction of the inactivated autogenous bone by liquid nitrogen combined with vascularized fibula with epiphyseal-preserving in our department between January 2019 and January 2020 were reviewed.

## 2. Methods

### 2.1. Patient characteristics

This study was approved by the Ethics Committee of Affiliated Tumor Hospital of Xinjiang Medical University and informed consent was obtained from all patients. Inclusion criteria: primary malignant tumor of bone near the knee joint; neoadjuvant chemotherapy is effective; the tumor did not invade the epiphyseal plate; the tumor did not invade important blood vessels and nerves; no infection. Exclusion criteria: pathological fracture; no limb preservation conditions; tumor invading epiphysis. There were 3 male and 2 female patients, the age range was from 8 to 14 years, with an average of 11.6 years. Distal femoral lesions were observed in 2 cases and proximal tibial lesions in 3 cases. All patients underwent X-ray, computed tomography, magnetic resonance imaging, and emission computed tomography examination, and a biopsy was performed after the examination. All cases were common osteosarcomas with no distant metastasis. According to the Enneking staging system, all cases were classified as stage IIB. The distance between the epiphyseal plate and the tumor was >1 cm in all cases. The magnetic resonance imaging image San Julian classification^[5]^ was applied to classify the lesions. Type I lesions are defined as a distance from the edge of the tumor to the epiphyseal plate >2 cm; for Type II the distance from the edge of the tumor to the epiphyseal plate <2 cm or adjacent; for Type III the epiphyseal plate is partially in contact with the tumor or invaded epiphysis. All cases were classified as San Julian I or San Julian II and the epiphysis could be preserved. All the patients were treated with preoperative neoadjuvant chemotherapy, surgery, and postoperative adjuvant chemotherapy. All lesions were sensitive to preoperative neoadjuvant chemotherapy, and no pathological fractures occurred during chemotherapy. The chemotherapy regimen consisted of cisplatin 100 mg/m^2^, adriamycin 80 mg/m^2^, methotrexate 12 g/m^2^, and ifosfamide 12 g/m^2^.

### 2.2. Surgical procedure

**Figure 1.**
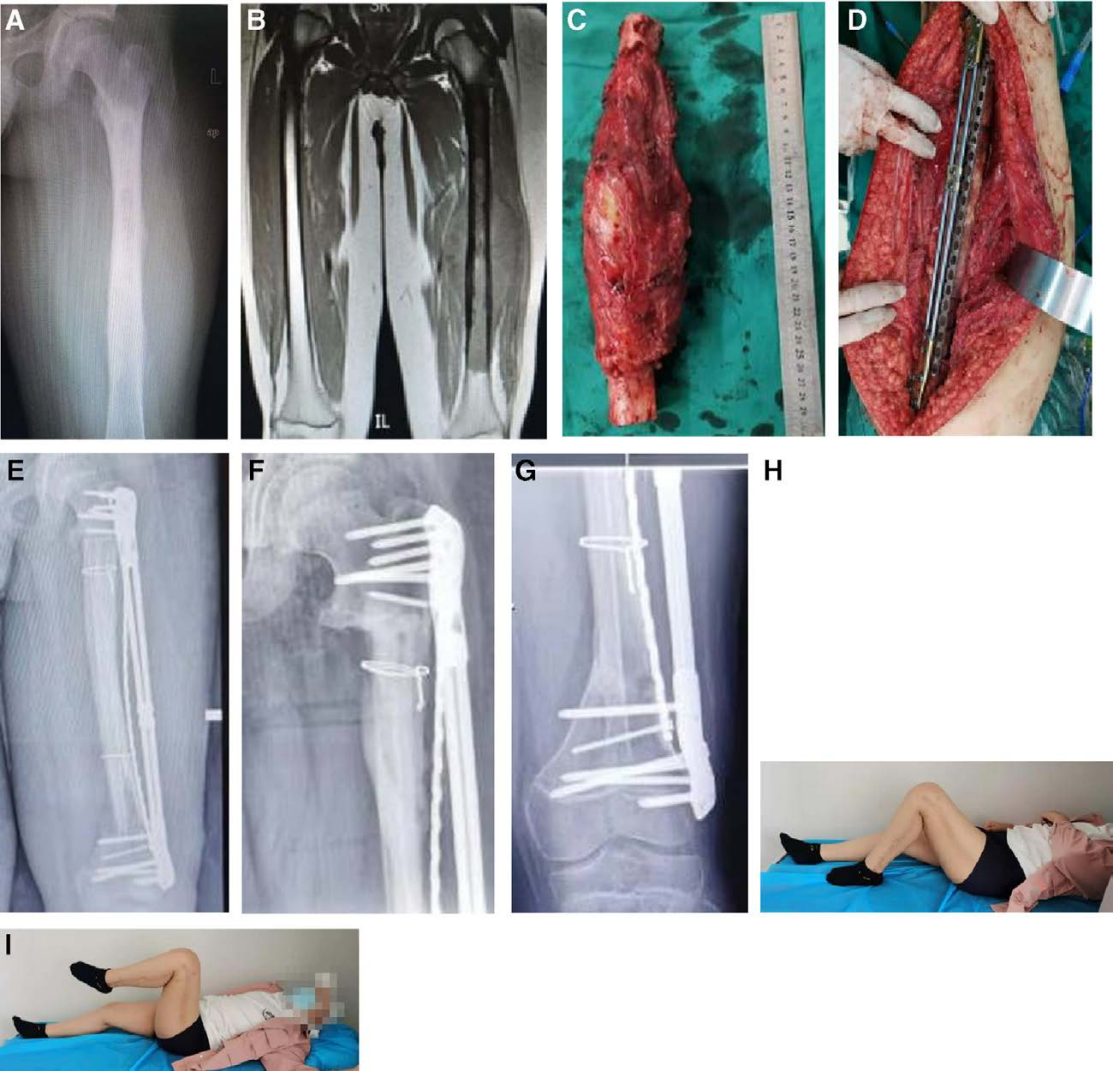
(A) The preoperative X-ray. (B) The preoperative MRI. (C) The focus of osteosarcoma in the middle femur was 30 cm long. (D) We anastomosed the vascularized fibula first, implanted the inactivated femur, and fixed it with 2 steel plates, finally. (E) Postoperative X-ray. (F and G) The X-ray showed 3 years after the operation. (H and I) The patient’s lower limb function was satisfactory. MRI = magnetic resonance imaging.

**Figure 2.**
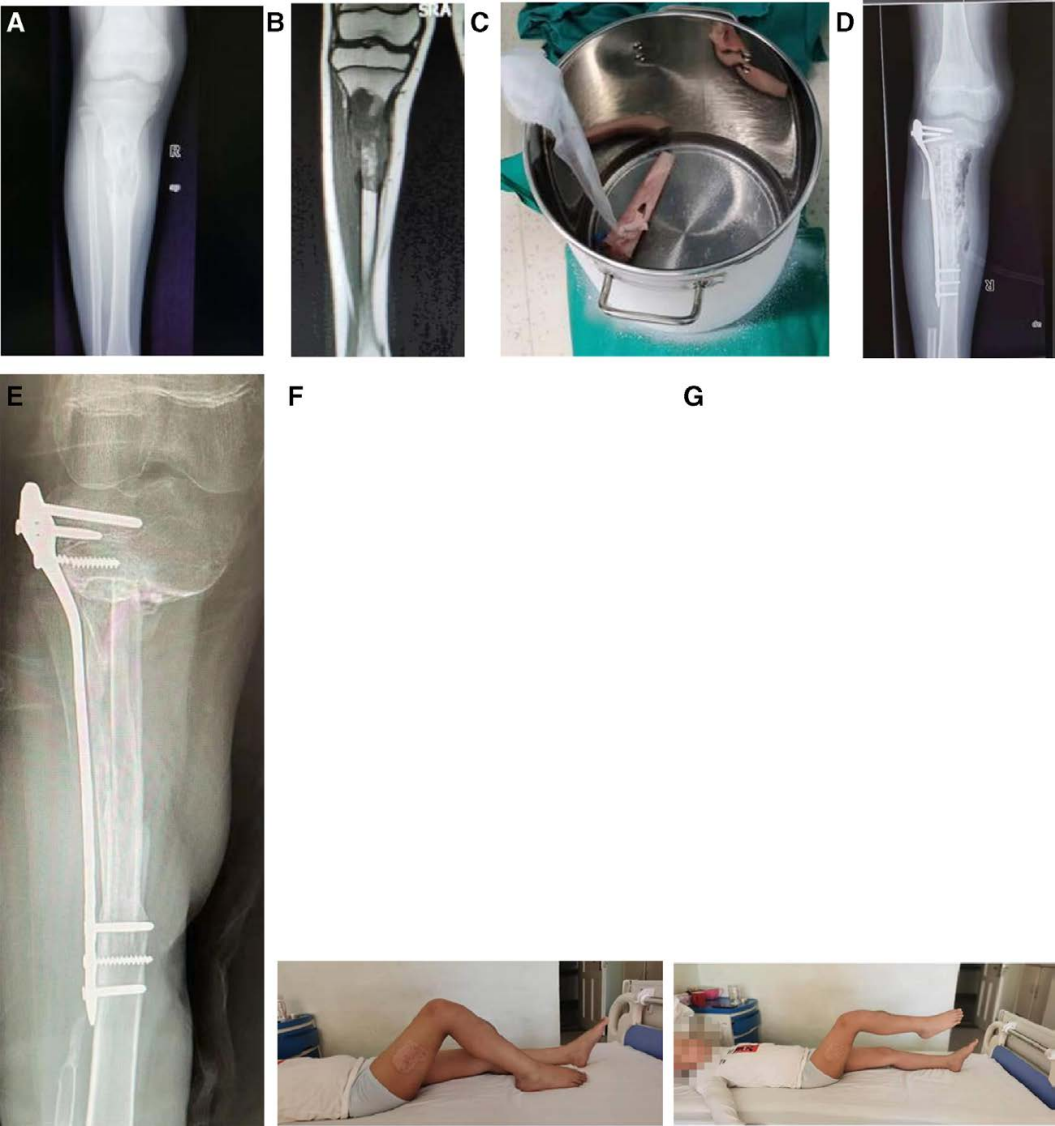
(A) The preoperative X-ray. (B) The preoperative MRI. (C) Autologous bone liquid nitrogen inactivation. (D) Postoperative X-ray. (E) X-ray showed 2 years after the operation. (F and G) The patient’s lower limb function was satisfactory. MRI = magnetic resonance imaging.

The surgical procedure involving the distal femoral tumor was as follows. The incision was located on the inner side of the thigh, and the puncture channel was removed. The quadriceps femoris tendon was preserved, the femoral artery and vein were dissociated, and the intermediate femoral muscle and part of the medial femoral muscle were removed together with the tumor. The proximal osteotomy line was 3 cm away from the tumor and the distal osteotomy line was 0.5 to 2 cm away from the epiphyseal line. In 1 patient with a tumor focus of approximately 30 cm in length and close to the epiphysis of the proximal and distal ends of the femur, the proximal osteotomy line was 0.5 cm away from the epiphysis of the greater and lesser trochanter and the distal osteotomy line was 2 cm away from the epiphysis. No tumor was identified in the proximal and distal medullary cavity specimens. The tumor and soft tissue on the surface of the tumor bone and in the medullary cavity were removed, the reactive bone was removed and only the normal bone cortex was retained. Liquid nitrogen was inactivated for 30 minutes and then rewarmed for 40 minutes. The fibula with the vascular pedicle was harvested, and the length was 2 to 3 cm longer than the tumor segment. The vascularized fibula was inserted into the femur and anastomosed with the branch of the deep femoral artery, and then fixed with double plates. In 1 case the femoral inactivated bone had a large curve and as the vascularized fibula could not be sleeved into the femoral medullary cavity, the inactivated bone was cut longitudinally. We first implanted the fibula. Then, the 2 halves of the femoral cortex were caged back to wrap the fibula, bound with a steel wire, and fixed with 2 steel plates (Fig. 1). An anterior medial tibial incision was used for proximal tibial tumors, and the puncture channel was removed at the same time. Osteotomy was performed at a distance of 3 cm from the tumor at the distal end and 0.5 to 2 cm from the epiphyseal line. The patellar ligament can be retained in part or completely. The process of tumor bone treatment was similar to that in the femur. The ipsilateral vascularized fibula was inserted into the tibial medullary cavity by pushing or rotating the fibula segment in 2 patients, and the contralateral vascularized fibula was embedded into the autologous bone and then vascular anastomosis was performed in 1 patient. After the placement of a single tibial plate, the medial gastrocnemius myocutaneous flap was rotated to cover the proximal tibia, and then the patellar ligament was reconstructed (Fig. 2).

### 2.3. Postoperative treatment

The patients were provided with guidance for exercises involving muscle contraction and knee flexion and extension. Postoperative adjuvant chemotherapy was started 2 weeks after surgery, and radiographs were performed every 3 months after surgery. When bone healing was visible on the radiographs, weight-bearing exercises were commenced. The presence of a continuous callus on the radiograph with the disappearance of the fracture line at the connecting part indicated complete healing and the patients progressed to full weight-bearing.

## 3. Results

Three male and 2 female patients aged from 8 to 14 years, with an average age of 11.6 years, were included in this study. In 2 cases, the lesion was located in the femur, while in 3 the lesion was located in the tibia. All cases involved common osteosarcoma. The average length of the bone resection was 18 cm (12–30 cm). The operation time was approximately 6 to 10 hours, and the amount of intraoperative bleeding was approximately 600 to 1000 mL. All patients were reviewed every 3 months and followed up for 24 to 36 months, and underwent postoperative adjuvant chemotherapy as described above. No tumor recurrence and no distant metastasis were observed, and all patients survived to the last date of follow-up. The average duration of bone healing was 5.2 months (3–8 months). In the last follow-up, knee flexion was >90° in 4 cases and 50 to 60° in 1 case. In 2 cases, surgery achieved equal length of the lower limbs, in 1 case shortening ≤1 cm of the affected limb was observed, and in a further case shortening of 2 cm was observed. The average Muscle and Skeletal Tumor Society score was 24.2. No stress fractures occurred in the autologous bone or the fibula. One patient with tibial osteosarcoma had an incision dehiscent infection after surgery, and the incision healed after debridement (Table 1).

**Table 1:** Patient information and clinical treatment results.

	Age (yr)	Gender	Diagnosis	Location	Defect length	Bone-healing time	Limb length	Knee flexion	Complication	MSTS
1	10	Male	OS	Tibia	15 cm	6 mo	Shorten 1 cm	>90°	Infect	25
2	8	Female	OS	Femur	12 cm	8 mo	Shorten 0.6 cm	>90°	No	20
3	14	Male	OS	Femur	30 cm	3 mo	Equal	>90°	No	26
4	14	Female	OS	Tibia	17 cm	4 mo	Equal	>90°	No	24
5	12	Male	OS	Tibia	16 cm	5 mo	Shorten 2 cm	50–60°	No	26

MSTS = Muscle and Skeletal Tumor Society, OS = osteosarcoma.

## 4. Discussion

The goal of limb salvage surgery for osteosarcoma is to achieve complete extended tumor resection as well as good limb function. With the development of chemotherapy, imaging, and surgical technology, periarticular osteosarcoma of the knee that does not invade the epiphysis can be accurately removed, preserving joint function but also retaining growth potential and reducing length discrepancy between the lower limbs.

Prosthesis reconstruction after tumor resection achieved good results in the early stages. However, with the improvement in osteosarcoma survival rates, the 10-year life of the tumor prosthesis was <50%.^[6]^ With time, the incidence of prosthesis loosening and fractures and the prosthesis revision rate have increased. Tumor prosthesis revision is more challenging than revision of an ordinary joint prosthesis, and decreased function and patient satisfaction after revision of the tumor prosthesis have been reported.

A large allogeneic bone provides initial mechanical strength, although complications of this method include infection, fracture, and bone nonunion. Low osteogenic activity, slow bone healing, or even nonunion have been reported with large allogenic bone after transplantation.^[7]^ Furthermore, matching the allogenic bone is challenging. Tsuchiya et al^[8]^ reported on 19 patients with large bone defects after tumor resection. The patients underwent external fixation by bone transfer combined with shortening and distraction osteogenesis by intramedullary nails. Common complications of bone transfer include nonunion, unequal length of the lower limbs, nail canal infection, and angular deformities. The most common complication was nail canal infection, with an incidence of 4 to 13%, while the incidence of bone nonunion was low. Masquelet described the induced membrane technology to reconstruct bone defects.^[9]^ However, the authors reported a 10 to 49% incidence of complications such as infection, recurrence, nonunion or malunion, and refracture. Furthermore, difficulty in removing the bone cement in secondary surgery, which may further increase the bone defect, damage the induced membrane, and result in slow corticalization after clinical healing, was also reported.^[10]^

Another reconstruction method is the inactivation of autologous bone replantation, which has the advantage of anatomical matching and is a simple procedure with relatively low costs and no need for large bone bank support. Under certain conditions, autologous bone inactivation replantation is a suitable reconstruction method that includes high-temperature and high-pressure inactivation, pasteurization, external irradiation, absolute ethanol inactivation, and liquid nitrogen inactivation.^[11]^ In 2005, Tsuchiya et al^[12]^ reported that liquid-nitrogen-inactivated autologous bone reconstruction achieved good results after tumor resection. Since 2015, Li et al^[13]^ have applied cryogenic liquid nitrogen inactivation technology for the resection and repair of bone defects after malignant bone tumors. The local recurrence rate was 5% (1/21), which was significantly lower than that for absolute alcohol inactivation and replantation (26.7%, 51/191). Alcohol could only inactivate the thin lateral cortex and medullary cortex tumors, while deep tumor cell death was achieved over 4 days of treatment. Internal fixation destroys the surface of the inactivated bone. Angiogenesis of tumor cells in deep bones results in recurrence. In the process of liquid nitrogen inactivation, after the autologous bone was placed in liquid nitrogen for 15 minutes, the medullary cavity temperature steadily decreased to below −150°C, which ensured the death of all tumor cells in inactivated bone.^[14]^ The results showed that liquid nitrogen inactivation of primary malignant tumors was safe. We used the liquid nitrogen inactivation technique and no tumor recurrence was observed in the 5 cases at the last follow-up.

Inactivated bone healing is defined as healing between the inactivated bone and the host bone. The inactivation process kills all cells in the bone; therefore, inactivated bone healing requires a long time and has a high risk of bone nonunion. This process is similar to the healing of a large allogeneic bone. One study reported a 17.3% (33/191) replantation nonunion rate after absolute alcohol inactivation,^[11]^ while another study reported that 38 (23.8%) of 164 patients who underwent large-segment allograft did not achieve healing.^[15]^ Li et al^[13]^ found that healing was achieved in 94.4% of patients (n = 21) with autogenous bone inactivated with liquid nitrogen. Igarashi et al^[16]^ reported that 6 (16.6%) of 36 patients who received autogenous bone liquid nitrogen inactivated bone transplantation did not achieve healing. Evidence indicates that the healing rate of liquid nitrogen-inactivated bone was higher than that of allogeneic bone transplantation and alcohol inactivation.^[17]^ Takata et al^[18]^ found that frozen autologous grafts treated with liquid nitrogen could better preserve bone morphogenesis and maintain bone induction and bone conductivity than those treated with autoclaving and pasteurization in animal experiments.

Although the healing rate of liquid nitrogen-inactivated bone is high, non-healing may occur in some cases and there is a risk of fracture. Capanna et al^[19]^ reported a segmental defect of long bone after tumor resection combined with vascularized fibula and large allogeneic bone. Noguchi and Ozaki et al^[20,21]^ described the application of vascularized fibula combined with pasteurized autologous bone implantation in osteosarcoma. The combination of these technologies obtains good clinical efficacy and a low incidence of complications. Autologous bone provides anatomical matching and initial mechanical stability. The osteogenic potential of vascularized fibula improved functional healing and reduced the risk of bone nonunion. Healing of the fibula provides late mechanical stability. Soucacos et al^[22]^ reviewed 18 cases of vascularized fibula transplantation to reconstruct large bone defects, with a healing rate of 92% and an average healing time of 3 months. In our study, 5 patients were treated with autologous bone liquid nitrogen inactivation combined with vascularized fibula transplantation. The ipsilateral vascularized fibula was transplanted and reconstructed in 2 patients with femur lesions. The ipsilateral fibula was resected and moved to the tibial defect without anastomosing blood vessels in the 2 patients with tibial lesions. This was a relatively simple procedure and the blood supply was generally not affected. However, this may result in a discrepancy in postoperative limb length and there is an increased risk of postoperative fracture. Vascularized fibula transplantation on the contralateral side of the tibia in 1 patient with tibial involvement could support and maintain the length of the limb, although there was a risk of vascular occlusion affecting the blood supply of the fibula segment. The average bone-healing time was 5.2 months. Better limb function was associated with earlier bone healing.

Retaining the epiphysis may preserve the growth potential of the limbs, reduce the length discrepancy between the affected limb and the healthy limb, and improve limb function. Manfrini et al^[23]^ followed up 6 patients with osteosarcoma with epiphyseal preservation and found that the average length of the affected limb was 2.2 cm (0.5–3.3 cm) shorter than that of the healthy side, which had little effect on walking function and did not require specific treatment. The flexion and extension function of the affected knee joint could be restored to 95% of normal function, and there was no joint instability. In the last follow-up of our study, knee flexion was >90° in 4 cases and 50 to 60° in 1 case. There was no valgus deformity in the affected limb, with equal length of both lower limbs in 2 cases and shortening ≤1 cm in 1 case. In 1 patient with a 2 cm shortening of the affected limb, walking function was minimally impaired. The average Muscle and Skeletal Tumor Society was 24.2, and the patients were satisfied with the treatment results.

Manfrini^[23]^ ported that screw fixation of the epiphysis had little effect on the growth of the epiphysis and would not seriously affect limb growth. Aponte^[24]^ found that postoperative refractures were mostly located in the metaphysis of the distal femur, which was related to the area that was not covered by internal fixation. It was suggested that the entire allograft should be covered with a long steel plate to provide extra cortical support. Muscolo et al^[25]^ postulated that the plate and screw would exert more pressure on the inactivated autologous bone interface and provide better stability. Kreig et al^[26]^ stated that simple screw fixation is more likely to lead to fracture than plate or intramedullary nail fixation. Therefore, in metaphyseal tumor resection, the use of a locked intramedullary nail or steel plate at the metaphyseal epiphyseal junction is recommended. We used steel plate fixation. Considering that there was sufficient soft tissue on the femoral side, we usually used 2 steel plate fixations that could be completely covered. After tibial tumor resection, single-plate fixation is generally used as there is less soft tissue available. Distal or proximal screws of the steel plate were placed in the epiphysis. No limb valgus deformities were observed during the last follow-up.

Common complications after bone defect reconstruction include delayed wound healing and infection, with a reported incidence of 5 to 11%.^[16]^ One patient had an incision dehiscent infection that healed after debridement. Good soft-tissue repair can reduce wound complications. Particularly in tibial tumor surgery, the application of a medial gastrocnemius muscle flap turnover coverage steel plate may reduce the incidence of infection.

This study has some limitations. The number of patients was small, and the follow-up time was short. We will continue to follow up the current patients for long-term complications and limb function.

## 5. Conclusion

Recent follow-up results showed that the incidence of complications was low, and the fracture healing rate was high. The shortening of the affected limb was not obvious and did not affect the walking function. The postoperative functional recovery was satisfactory. Inactivation of autologous bone by liquid nitrogen combined with vascularized fibula transplantation with epiphyseal preservation was a reliable biological reconstruction method.

## Author contributions

**Investigation:** Yachao Sun, Maierdanjiang Maihemuti.

Project administration: Renbing Jiang.

Writing – original draft: Zhibing Dai.

Writing – review & editing: Zhibing Dai.
